# Supplementary figures and images for: Postprandial transfer of colostral extracellular vesicles and their protein and miRNA cargo in neonatal calves
Source: PLoS One. 2020 Feb 28;15(2):e0229606. doi: 10.1371/journal.pone.0229606 (PMC7048281; doi:10.1371/journal.pone.0229606)

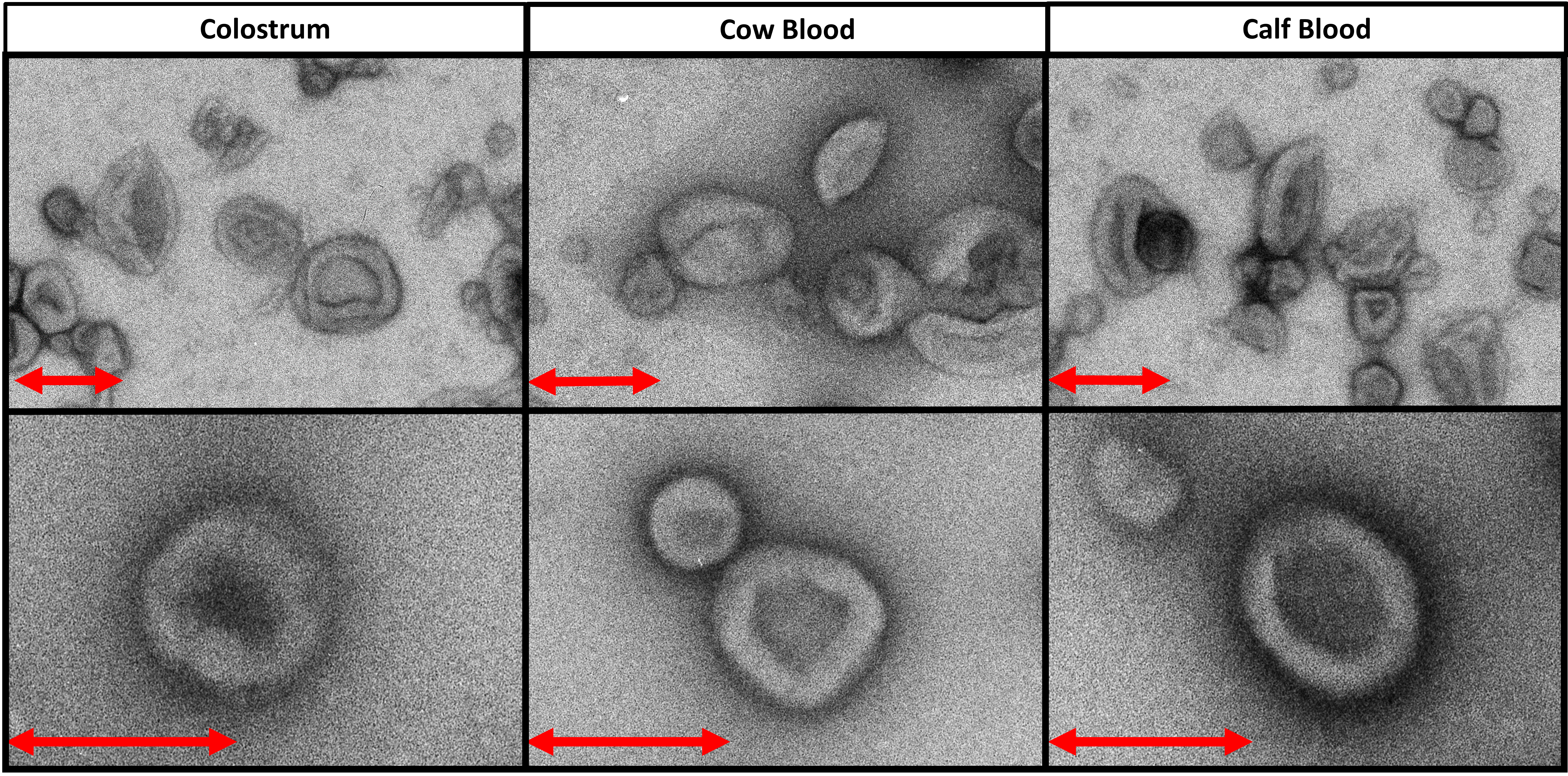

Supplement: S1 Fig — Images are representative for three separate biological replicates per sample group. No differences were observed for postprandial time points in calf blood EV. (TIFF) [file pone.0229606.s001.tiff]

EVs 40 + 50 % SDG – CNX, HSP70, BTN1A1

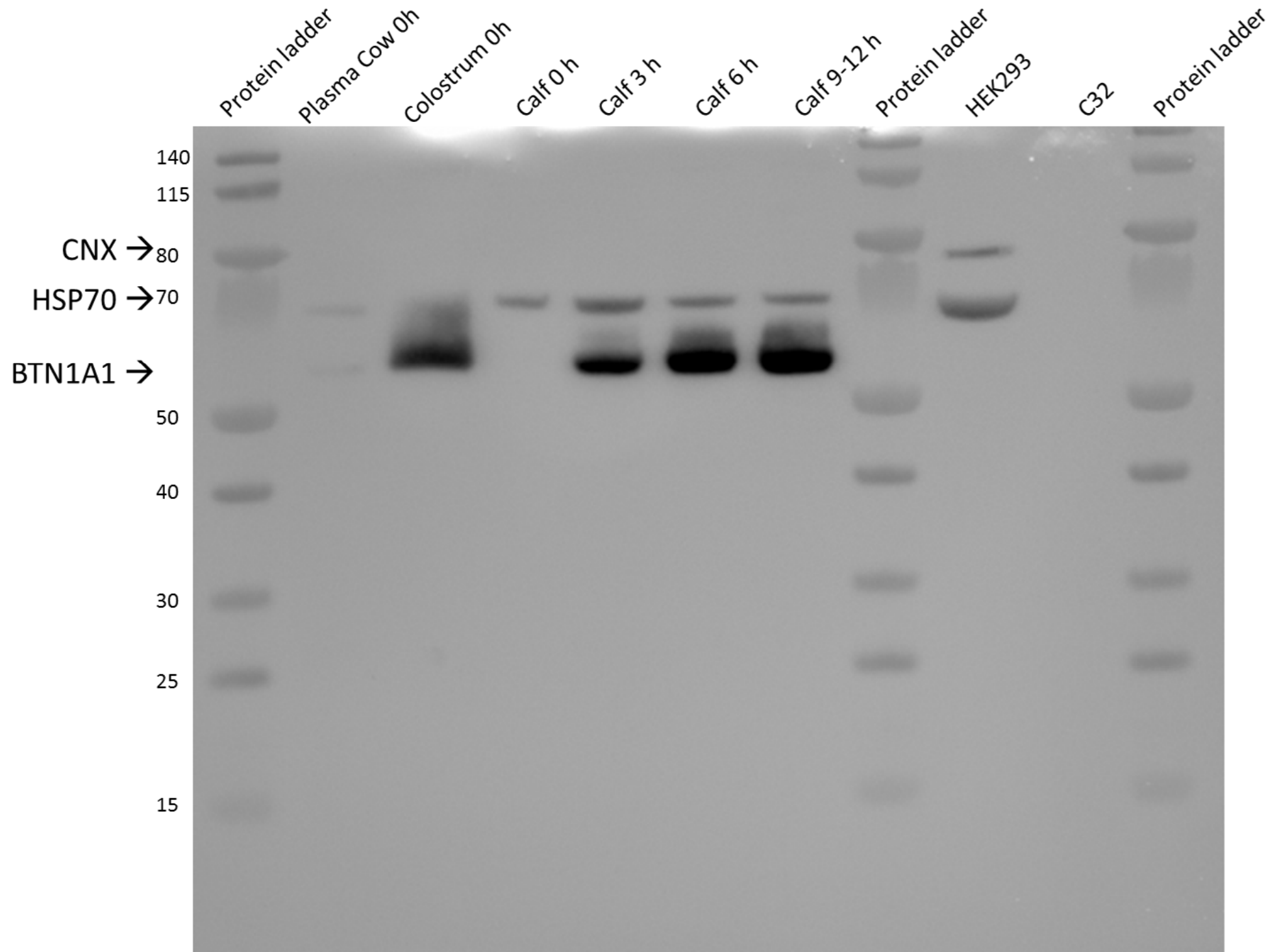

EVs 40 + 50 % SDG – MFGE8

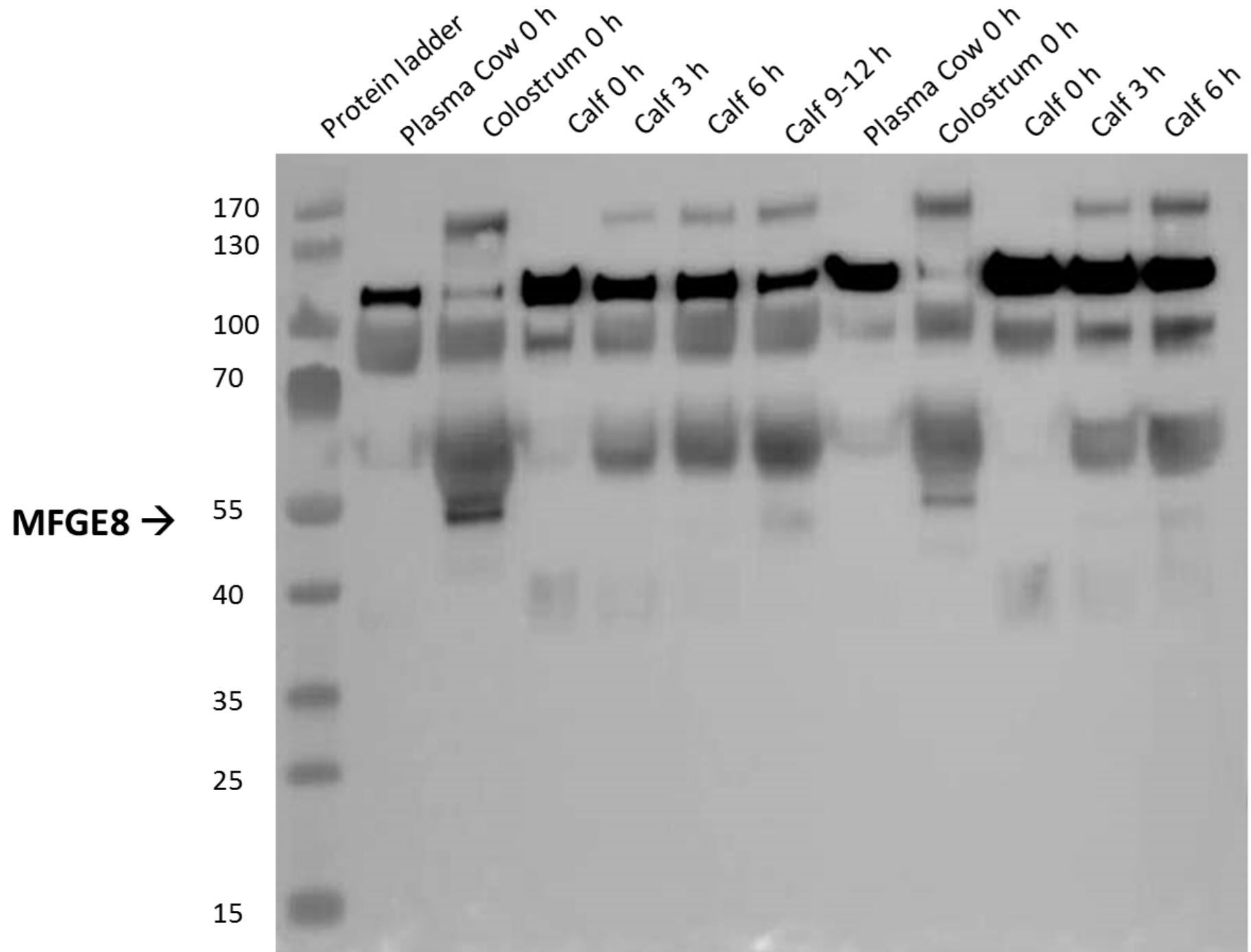

EVs 40 + 50 % SDG – CD63

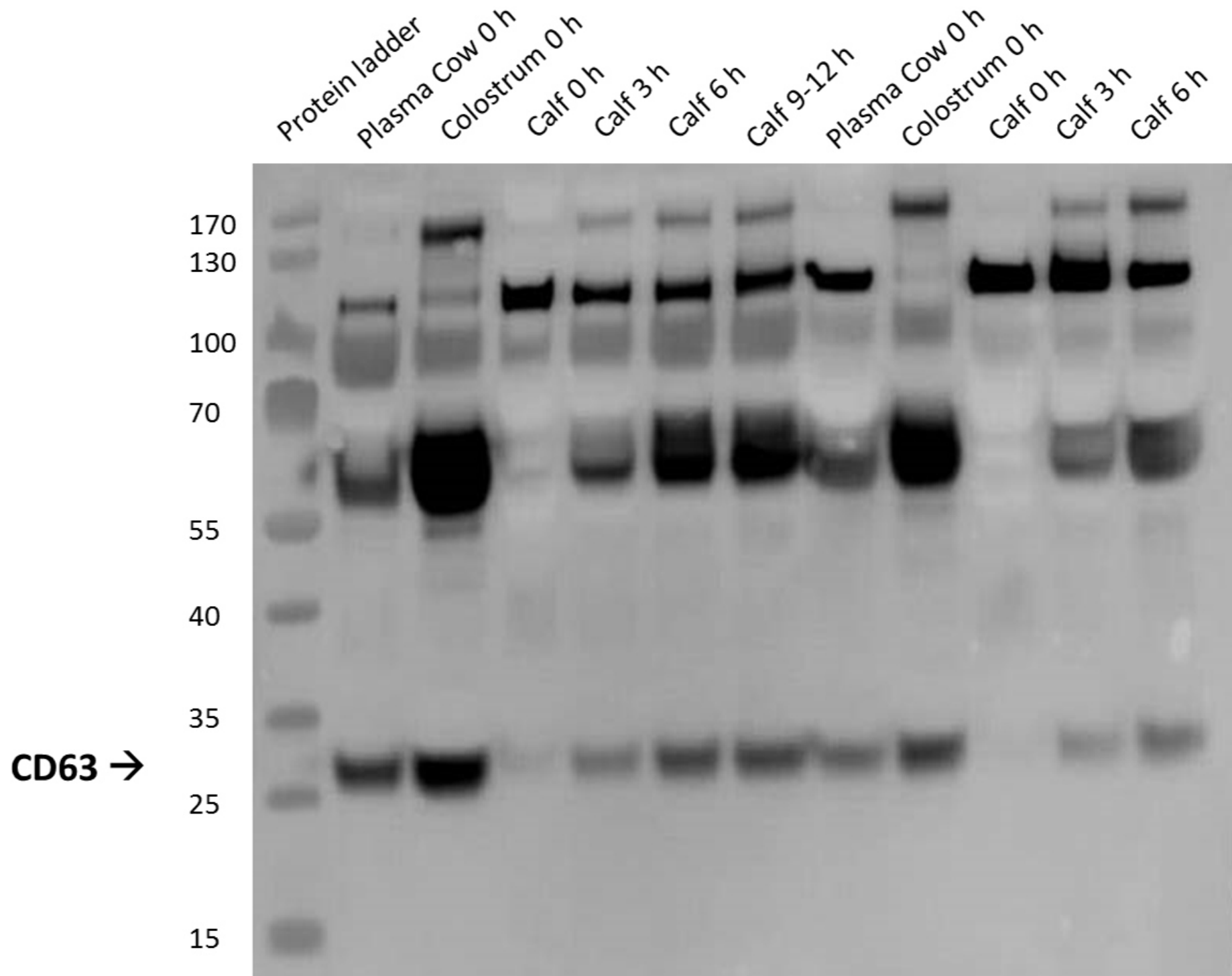

Supplement: S1 Raw images — (PDF) [file pone.0229606.s007.pdf]
