# Supplementary material for: Postprandial transfer of colostral extracellular vesicles and their protein and miRNA cargo in neonatal calves
Source: PLoS One. 2020 Feb 28;15(2):e0229606. doi: 10.1371/journal.pone.0229606 (PMC7048281; doi:10.1371/journal.pone.0229606)
Supplement: S1 Table — Measurements are summarized as mean values and standard deviations within a sampling group and size distributions are further characterized by giving the most frequent particle size. (DOCX) [file pone.0229606.s002.docx]

**S1 Table.** **Particle size and concentrations of EV suspensions as determined by NTA analysis.** Measurements are summarized as mean values and standard deviations within a sampling group and size distributions are further characterized by giving the most frequent particle size.

|  | **SDG** | **Particle size in nm** | | | **Particles per ml** | |
| --- | --- | --- | --- | --- | --- | --- |
|  |  | **Mean** | **Mode** | **SD** | **Mean** | **SD** |
| **Cow blood EV** | **30%** | 139.3 | 129.4 | 57.2 | 2.97E+09 | 3.15E+08 |
|  | **40-50%** | 167.8 | 112.4 | 97.0 | 2.07E+09 | 5.17E+08 |
| **Colostrum EV** | **30%** | 129.6 | 98.3 | 62.7 | 2.42E+09 | 6.67E+08 |
|  | **40-50%** | 132.3 | 110.5 | 61.5 | 4.59E+10 | 4.25E+09 |
| **Calf EV 0h** | **30%** | 148.9 | 124.2 | 56.5 | 3.03E+08 | 1.66E+08 |
|  | **40-50%** | 154.7 | 134.7 | 65.2 | 2.77E+09 | 7.14E+08 |
| **Calf EV 3h** | **30%** | 145.7 | 125.2 | 59.1 | 2.19E+08 | 4.47E+07 |
|  | **40-50%** | 155.9 | 138.5 | 57.3 | 1.09E+10 | 1.92E+09 |
| **Calf EV 6h** | **30%** | 142.7 | 121.8 | 50.2 | 3.39E+08 | 2.39E+08 |
|  | **40-50%** | 160.3 | 142.9 | 56.9 | 1.14E+10 | 1.95E+09 |
| **Calf EV 9-12h** | **30%** | 142.3 | 122.1 | 53.4 | 3.70E+08 | 1.39E+08 |
|  | **40-50%** | 161.1 | 142.1 | 57.8 | 1.97E+10 | 7.33E+09 |
