# Supplementary material for: Postprandial transfer of colostral extracellular vesicles and their protein and miRNA cargo in neonatal calves
Source: PLoS One. 2020 Feb 28;15(2):e0229606. doi: 10.1371/journal.pone.0229606 (PMC7048281; doi:10.1371/journal.pone.0229606)
Supplement: S2 Table — Short: smaller than 16 nt, No Adaptor: 50 nt long with no detectable adaptor sequence at 5’-end. Read numbers are given as mean values for each sample group. (DOCX) [file pone.0229606.s003.docx]

**S2 Table.** **Distribution of raw read counts on small ncRNA species and inappropriate length classes**. Short: smaller than 16 nt, No Adaptor: 50 nt long with no detectable adaptor sequence at 5’-end. Read numbers are given as mean values for each sample group.

|  | **Colostrum** | **Colostrum Cells** | **Colostrum EV** | **Cow Blood** | **Calf Blood** | **Calf Cells** | **Calf EV** |
| --- | --- | --- | --- | --- | --- | --- | --- |
| **No Adaptor** | 1.35E+02 | 3.80E+01 | 1.27E+02 | 6.40E+01 | 6.40E+01 | 2.10E+01 | 1.80E+01 |
| **Short** | 1.63E+06 | 2.34E+05 | 1.34E+06 | 3.75E+05 | 2.86E+05 | 5.18E+04 | 1.36E+05 |
| **Unmapped** | 1.41E+06 | 3.78E+06 | 1.32E+06 | 5.97E+05 | 8.53E+05 | 4.21E+05 | 3.79E+06 |
| **rRNA** | 3.32E+05 | 1.46E+06 | 2.44E+05 | 7.06E+04 | 4.61E+04 | 1.15E+05 | 3.25E+05 |
| **snRNA** | 5.85E+03 | 9.84E+04 | 2.40E+03 | 2.88E+03 | 2.42E+03 | 3.06E+03 | 9.92E+02 |
| **snoRNA** | 3.20E+04 | 8.58E+04 | 8.22E+03 | 4.44E+04 | 3.08E+04 | 4.11E+04 | 1.02E+03 |
| **tRNA** | 1.46E+06 | 5.99E+06 | 7.18E+05 | 2.20E+04 | 3.27E+04 | 2.89E+04 | 6.67E+04 |
| **miRNA/isomiR** | 2.06E+06 | 1.73E+06 | 3.18E+06 | 6.70E+06 | 6.65E+06 | 4.98E+06 | 6.83E+05 |
| **Total library size** | 6.93E+06 | 1.34E+07 | 6.81E+06 | 7.81E+06 | 7.90E+06 | 5.64E+06 | 5.01E+06 |
