# Supplementary material for: Postprandial transfer of colostral extracellular vesicles and their protein and miRNA cargo in neonatal calves
Source: PLoS One. 2020 Feb 28;15(2):e0229606. doi: 10.1371/journal.pone.0229606 (PMC7048281; doi:10.1371/journal.pone.0229606)
Supplement: S3 Table — Green background indicates up-regulation compared to calf EV 0h samples while red background highlights down-regulation. Expression changes without significancy are denoted by n.s. (DOCX) [file pone.0229606.s004.docx]

**S3 Table.** **log2 fold-changes of significantly regulated canonical miRNAs in colostral and postprandial calf blood EV compared to pre-feeding calf blood EVs**. Green background indicates up-regulation compared to calf EV 0h samples while red background highlights down-regulation. Expression changes without significancy are denoted by n.s.

|  |  | **Postprandial Time Points** | | | |
| --- | --- | --- | --- | --- | --- |
| **Canonical miRNA** | **Colostrum EVs** | **1h** | **3h** | **6h** | **9-12 h** |
| **bta-let-7a-5p** | 1.40 | n.s. | n.s. | n.s. | n.s. |
| **bta-let-7b** | 2.73 | n.s. | n.s. | n.s. | n.s. |
| **bta-let-7c** | 1.85 | n.s. | n.s. | n.s. | 1.25 |
| **bta-let-7e** | 1.77 | n.s. | n.s. | n.s. | n.s. |
| **bta-let-7f** | 1.37 | n.s. | n.s. | n.s. | n.s. |
| **bta-let-7g** | 1.19 | n.s. | n.s. | n.s. | n.s. |
| **bta-miR-100** | -3.20 | n.s. | n.s. | 1.17 | 1.98 |
| **bta-miR-103** | 2.63 | n.s. | n.s. | n.s. | n.s. |
| **bta-miR-106a** | 2.66 | n.s. | n.s. | n.s. | n.s. |
| **bta-miR-106b** | 2.43 | n.s. | n.s. | n.s. | n.s. |
| **bta-miR-107** | 2.71 | n.s. | n.s. | n.s. | n.s. |
| **bta-miR-10a** | -6.09 | n.s. | n.s. | n.s. | n.s. |
| **bta-miR-10b** | -11.37 | n.s. | n.s. | n.s. | n.s. |
| **bta-miR-122** | -11.97 | n.s. | n.s. | n.s. | n.s. |
| **bta-miR-125b** | 1.31 | n.s. | n.s. | n.s. | n.s. |
| **bta-miR-126-3p** | -10.73 | n.s. | n.s. | n.s. | n.s. |
| **bta-miR-126-5p** | -10.05 | n.s. | 1.58 | 1.71 | n.s. |
| **bta-miR-127** | -9.60 | n.s. | n.s. | n.s. | n.s. |
| **bta-miR-128** | -4.45 | n.s. | n.s. | n.s. | n.s. |
| **bta-miR-1306** | -3.92 | n.s. | n.s. | n.s. | n.s. |
| **bta-miR-1307** | -1.44 | n.s. | n.s. | n.s. | n.s. |
| **bta-miR-134** | -8.99 | n.s. | n.s. | n.s. | n.s. |
| **bta-miR-1343-3p** | -2.44 | n.s. | n.s. | n.s. | n.s. |
| **bta-miR-1388-5p** | -2.89 | n.s. | 1.85 | 1.96 | 2.26 |
| **bta-miR-139** | -3.36 | n.s. | n.s. | n.s. | n.s. |
| **bta-miR-141** | 9.05 | n.s. | 4.19 | 4.46 | 4.48 |
| **bta-miR-142-3p** | -5.09 | n.s. | n.s. | n.s. | n.s. |
| **bta-miR-142-5p** | -3.36 | n.s. | n.s. | n.s. | n.s. |
| **bta-miR-143** | -3.56 | n.s. | n.s. | n.s. | n.s. |
| **bta-miR-144** | -3.23 | n.s. | n.s. | n.s. | n.s. |
| **bta-miR-1468** | -5.78 | n.s. | n.s. | n.s. | n.s. |
| **bta-miR-148a** | 3.30 | n.s. | n.s. | n.s. | n.s. |
| **bta-miR-150** | -7.07 | n.s. | n.s. | n.s. | n.s. |
| **bta-miR-151-3p** | -1.55 | n.s. | n.s. | n.s. | n.s. |
| **bta-miR-151-5p** | 2.71 | n.s. | n.s. | n.s. | n.s. |
| **bta-miR-152** | 1.95 | n.s. | n.s. | n.s. | n.s. |
| **bta-miR-154c** | -7.54 | n.s. | n.s. | n.s. | n.s. |
| **bta-miR-181a** | 2.42 | n.s. | n.s. | n.s. | n.s. |
| **bta-miR-181b** | 1.43 | n.s. | n.s. | n.s. | n.s. |
| **bta-miR-182** | 8.95 | n.s. | 3.28 | 3.41 | 3.79 |
| **bta-miR-183** | 9.33 | n.s. | 3.51 | 3.89 | 4.45 |
| **bta-miR-185** | -2.22 | n.s. | n.s. | n.s. | n.s. |
| **bta-miR-192** | -2.25 | 1.84 | 4.22 | 4.48 | 5.05 |
| **bta-miR-194** | n.s. | 2.26 | 5.20 | 5.54 | 5.58 |
| **bta-miR-199a-3p** | -6.94 | n.s. | n.s. | n.s. | n.s. |
| **bta-miR-199a-5p** | -5.38 | n.s. | n.s. | n.s. | 1.63 |
| **bta-miR-199b** | -6.52 | n.s. | n.s. | n.s. | n.s. |
| **bta-miR-199c** | -6.69 | n.s. | n.s. | n.s. | n.s. |
| **bta-miR-19a** | 1.54 | n.s. | n.s. | n.s. | n.s. |
| **bta-miR-19b** | 4.30 | n.s. | n.s. | n.s. | n.s. |
| **bta-miR-200a** | 9.91 | 1.91 | 4.32 | 4.55 | 5.02 |
| **bta-miR-200b** | 8.57 | 2.27 | 4.52 | 4.90 | 5.41 |
| **bta-miR-200c** | 7.07 | 2.06 | 4.30 | 4.74 | 5.16 |
| **bta-miR-204** | -9.84 | n.s. | n.s. | n.s. | 1.89 |
| **bta-miR-20a** | 2.17 | n.s. | n.s. | n.s. | n.s. |
| **bta-miR-20b** | 1.62 | n.s. | n.s. | n.s. | n.s. |
| **bta-miR-21-5p** | 1.67 | n.s. | n.s. | n.s. | n.s. |
| **bta-miR-210** | 1.26 | n.s. | n.s. | n.s. | n.s. |
| **bta-miR-215** | -2.28 | 2.57 | 4.90 | 5.15 | 5.66 |
| **bta-miR-22-3p** | 2.90 | n.s. | n.s. | n.s. | n.s. |
| **bta-miR-221** | -3.12 | n.s. | n.s. | n.s. | n.s. |
| **bta-miR-222** | -4.04 | n.s. | n.s. | n.s. | n.s. |
| **bta-miR-223** | n.s. | n.s. | n.s. | 2.67 | n.s. |
| **bta-miR-224** | -1.85 | n.s. | n.s. | n.s. | n.s. |
| **bta-miR-2284j** | 9.05 | n.s. | n.s. | n.s. | n.s. |
| **bta-miR-2284x** | 1.01 | n.s. | n.s. | n.s. | n.s. |
| **bta-miR-2284y** | 2.74 | n.s. | n.s. | n.s. | n.s. |
| **bta-miR-2285aa** | -2.16 | n.s. | n.s. | n.s. | n.s. |
| **bta-miR-2285b** | -2.94 | n.s. | n.s. | n.s. | n.s. |
| **bta-miR-2285t** | 8.59 | n.s. | n.s. | n.s. | n.s. |
| **bta-miR-23a** | 3.89 | n.s. | n.s. | n.s. | n.s. |
| **bta-miR-23b-3p** | 4.49 | n.s. | n.s. | n.s. | n.s. |
| **bta-miR-24-3p** | 1.51 | n.s. | n.s. | n.s. | n.s. |
| **bta-miR-25** | -2.99 | n.s. | n.s. | n.s. | n.s. |
| **bta-miR-26a** | 1.88 | n.s. | n.s. | n.s. | n.s. |
| **bta-miR-26b** | 1.65 | n.s. | n.s. | n.s. | n.s. |
| **bta-miR-27a-3p** | 2.04 | n.s. | n.s. | n.s. | n.s. |
| **bta-miR-27a-5p** | n.s. | n.s. | n.s. | 1.36 | 1.33 |
| **bta-miR-27b** | 2.22 | n.s. | n.s. | n.s. | n.s. |
| **bta-miR-28** | 3.18 | n.s. | n.s. | 1.37 | 1.30 |
| **bta-miR-296-3p** | -1.53 | n.s. | n.s. | -1.16 | n.s. |
| **bta-miR-29a** | 2.55 | n.s. | n.s. | n.s. | n.s. |
| **bta-miR-30a-5p** | 6.39 | n.s. | n.s. | n.s. | n.s. |
| **bta-miR-30b-5p** | 6.02 | n.s. | n.s. | 2.10 | n.s. |
| **bta-miR-30f** | 5.63 | n.s. | n.s. | 1.04 | 1.13 |
| **bta-miR-32** | 3.80 | n.s. | n.s. | n.s. | n.s. |
| **bta-miR-323** | -8.48 | n.s. | n.s. | n.s. | n.s. |
| **bta-miR-328** | -1.54 | n.s. | n.s. | n.s. | n.s. |
| **bta-miR-330** | -4.21 | n.s. | -1.08 | -1.15 | -1.03 |
| **bta-miR-3432a** | -2.77 | n.s. | n.s. | n.s. | n.s. |
| **bta-miR-34a** | 9.52 | n.s. | n.s. | n.s. | n.s. |
| **bta-miR-361** | 1.60 | n.s. | n.s. | n.s. | n.s. |
| **bta-miR-362-5p** | 4.86 | n.s. | n.s. | n.s. | n.s. |
| **bta-miR-363** | 5.20 | n.s. | n.s. | n.s. | n.s. |
| **bta-miR-365-3p** | 2.56 | n.s. | n.s. | n.s. | n.s. |
| **bta-miR-370** | -8.46 | n.s. | n.s. | n.s. | n.s. |
| **bta-miR-374a** | 1.65 | n.s. | n.s. | n.s. | n.s. |
| **bta-miR-374b** | 4.46 | n.s. | n.s. | 1.51 | n.s. |
| **bta-miR-378** | n.s. | n.s. | n.s. | 1.66 | 2.35 |
| **bta-miR-378c** | 1.28 | n.s. | 1.79 | 2.03 | 2.51 |
| **bta-miR-378d** | 2.23 | n.s. | n.s. | n.s. | n.s. |
| **bta-miR-379** | -8.17 | n.s. | n.s. | n.s. | n.s. |
| **bta-miR-381** | -5.17 | n.s. | n.s. | n.s. | n.s. |
| **bta-miR-382** | -11.55 | n.s. | n.s. | n.s. | n.s. |
| **bta-miR-409a** | -7.77 | n.s. | n.s. | n.s. | n.s. |
| **bta-miR-411a** | -6.86 | n.s. | n.s. | n.s. | n.s. |
| **bta-miR-411c-5p** | -8.15 | n.s. | n.s. | n.s. | n.s. |
| **bta-miR-423-5p** | -2.76 | n.s. | n.s. | n.s. | n.s. |
| **bta-miR-425-3p** | 1.03 | n.s. | n.s. | n.s. | n.s. |
| **bta-miR-429** | 11.07 | n.s. | n.s. | n.s. | n.s. |
| **bta-miR-432** | -9.91 | n.s. | n.s. | n.s. | n.s. |
| **bta-miR-433** | -9.74 | n.s. | n.s. | n.s. | n.s. |
| **bta-miR-451** | -5.52 | n.s. | n.s. | n.s. | n.s. |
| **bta-miR-455-5p** | 2.78 | n.s. | n.s. | n.s. | n.s. |
| **bta-miR-484** | -1.80 | n.s. | n.s. | n.s. | n.s. |
| **bta-miR-486** | -9.51 | n.s. | n.s. | n.s. | n.s. |
| **bta-miR-493** | -8.44 | n.s. | n.s. | n.s. | n.s. |
| **bta-miR-494** | -5.99 | n.s. | n.s. | n.s. | n.s. |
| **bta-miR-495** | -5.63 | n.s. | n.s. | n.s. | n.s. |
| **bta-miR-502a** | 3.50 | n.s. | n.s. | n.s. | 2.04 |
| **bta-miR-502b** | 3.52 | n.s. | n.s. | n.s. | n.s. |
| **bta-miR-504** | -1.47 | n.s. | n.s. | n.s. | n.s. |
| **bta-miR-505** | -2.21 | n.s. | n.s. | n.s. | n.s. |
| **bta-miR-532** | 2.48 | n.s. | 1.22 | 1.42 | 2.02 |
| **bta-miR-543** | -10.84 | n.s. | n.s. | n.s. | n.s. |
| **bta-miR-574** | 3.93 | n.s. | n.s. | n.s. | 1.33 |
| **bta-miR-6119-5p** | 1.21 | n.s. | n.s. | n.s. | n.s. |
| **bta-miR-6120-3p** | -2.27 | n.s. | n.s. | n.s. | n.s. |
| **bta-miR-652** | 4.66 | n.s. | n.s. | n.s. | n.s. |
| **bta-miR-6524** | 2.81 | n.s. | n.s. | n.s. | n.s. |
| **bta-miR-6529a** | -2.08 | n.s. | n.s. | n.s. | n.s. |
| **bta-miR-660** | 3.03 | n.s. | 1.97 | 2.11 | 2.36 |
| **bta-miR-7** | 1.06 | n.s. | n.s. | n.s. | n.s. |
| **bta-miR-760-3p** | -1.07 | n.s. | n.s. | n.s. | n.s. |
| **bta-miR-9-5p** | -2.95 | n.s. | n.s. | n.s. | n.s. |
| **bta-miR-92a** | -1.56 | n.s. | n.s. | n.s. | n.s. |
| **bta-miR-92b** | -1.62 | n.s. | n.s. | n.s. | n.s. |
| **bta-miR-96** | 8.95 | n.s. | n.s. | n.s. | n.s. |
| **bta-miR-98** | 1.23 | n.s. | n.s. | n.s. | n.s. |
| **bta-miR-99a-5p** | 3.96 | n.s. | n.s. | n.s. | n.s. |
| **bta-miR-99b** | n.s. | n.s. | n.s. | n.s. | 1.08 |
