# Supplementary material for: Postprandial transfer of colostral extracellular vesicles and their protein and miRNA cargo in neonatal calves
Source: PLoS One. 2020 Feb 28;15(2):e0229606. doi: 10.1371/journal.pone.0229606 (PMC7048281; doi:10.1371/journal.pone.0229606)
Supplement: S5 Table — Analysis was performed on targets of human homologous miRNAs obtained from miRTarBase supported by strong experimental evidence (Reporter assay or Western blot). (DOCX) [file pone.0229606.s006.docx]

**S4 Table.** **Gene set enrichment of the top 20 KEGG pathways based on significantly up-regulated canonical miRNA in postprandial time points.** Analysis was performed on targets of human homologous miRNAs obtained from miRTarBase supported by strong experimental evidence (Reporter assay or Western blot).

| **KEGG Pathway** | **Adjusted p-value** | **Regulated miRNAs** |
| --- | --- | --- |
| **Insulin signaling pathway** | 2.64E-05 | hsa-let-7c-5p/hsa-miR-100-5p/hsa-miR-126-5p/hsa-miR-141-3p/hsa-miR-182-5p/hsa-miR-183-5p/hsa-miR-192-5p/hsa-miR-194-5p/hsa-miR-199a-5p/hsa-miR-200a-3p/hsa-miR-200b-3p/hsa-miR-200c-3p/hsa-miR-204-5p/hsa-miR-223-3p/hsa-miR-28-5p/hsa-miR-30b-5p/hsa-miR-374b-5p/hsa-miR-532-5p/hsa-miR-99b-5p |
| **TGF-beta signaling pathway** | 1.63E-04 | hsa-let-7c-5p/hsa-miR-100-5p/hsa-miR-126-5p/hsa-miR-141-3p/hsa-miR-182-5p/hsa-miR-183-5p/hsa-miR-192-5p/hsa-miR-194-5p/hsa-miR-199a-5p/hsa-miR-200a-3p/hsa-miR-200b-3p/hsa-miR-200c-3p/hsa-miR-204-5p/hsa-miR-215-5p/hsa-miR-223-3p/hsa-miR-28-5p/hsa-miR-30b-5p/hsa-miR-378a-5p |
| **Cytokine-cytokine receptor interaction** | 2.00E-04 | hsa-let-7c-5p/hsa-miR-100-5p/hsa-miR-126-5p/hsa-miR-141-3p/hsa-miR-192-5p/hsa-miR-194-5p/hsa-miR-199a-5p/hsa-miR-200a-3p/hsa-miR-200b-3p/hsa-miR-200c-3p/hsa-miR-204-5p/hsa-miR-215-5p/hsa-miR-223-3p/hsa-miR-27a-5p/hsa-miR-28-5p/hsa-miR-30b-5p/hsa-miR-374b-5p/hsa-miR-532-5p |
| **Signaling pathways regulating pluripotency of stem cells** | 2.01E-04 | hsa-let-7c-5p/hsa-miR-100-5p/hsa-miR-126-5p/hsa-miR-141-3p/hsa-miR-182-5p/hsa-miR-183-5p/hsa-miR-192-5p/hsa-miR-194-5p/hsa-miR-199a-5p/hsa-miR-200a-3p/hsa-miR-200b-3p/hsa-miR-200c-3p/hsa-miR-204-5p/hsa-miR-215-5p/hsa-miR-223-3p/hsa-miR-28-5p/hsa-miR-30b-5p/hsa-miR-374b-5p/hsa-miR-378a-5p/hsa-miR-99b-5p |
| **Choline metabolism in cancer** | 2.01E-04 | hsa-let-7c-5p/hsa-miR-100-5p/hsa-miR-141-3p/hsa-miR-182-5p/hsa-miR-192-5p/hsa-miR-194-5p/hsa-miR-199a-5p/hsa-miR-200a-3p/hsa-miR-200b-3p/hsa-miR-200c-3p/hsa-miR-204-5p/hsa-miR-223-3p/hsa-miR-27a-5p/hsa-miR-28-5p/hsa-miR-30b-5p/hsa-miR-374b-5p/hsa-miR-99b-5p |
| **Oocyte meiosis** | 2.01E-04 | hsa-let-7c-5p/hsa-miR-100-5p/hsa-miR-141-3p/hsa-miR-182-5p/hsa-miR-183-5p/hsa-miR-192-5p/hsa-miR-194-5p/hsa-miR-200a-3p/hsa-miR-200b-3p/hsa-miR-200c-3p/hsa-miR-204-5p/hsa-miR-223-3p/hsa-miR-28-5p/hsa-miR-30b-5p/hsa-miR-99b-5p |
| **Rap1 signaling pathway** | 3.12E-04 | hsa-let-7c-5p/hsa-miR-100-5p/hsa-miR-126-5p/hsa-miR-141-3p/hsa-miR-182-5p/hsa-miR-183-5p/hsa-miR-192-5p/hsa-miR-194-5p/hsa-miR-199a-5p/hsa-miR-200a-3p/hsa-miR-200b-3p/hsa-miR-200c-3p/hsa-miR-204-5p/hsa-miR-223-3p/hsa-miR-27a-5p/hsa-miR-28-5p/hsa-miR-30b-5p/hsa-miR-374b-5p/hsa-miR-99b-5p |
| **Jak-STAT signaling pathway** | 3.12E-04 | hsa-let-7c-5p/hsa-miR-100-5p/hsa-miR-126-5p/hsa-miR-141-3p/hsa-miR-182-5p/hsa-miR-183-5p/hsa-miR-192-5p/hsa-miR-194-5p/hsa-miR-199a-5p/hsa-miR-200a-3p/hsa-miR-200b-3p/hsa-miR-200c-3p/hsa-miR-204-5p/hsa-miR-223-3p/hsa-miR-27a-5p/hsa-miR-28-5p/hsa-miR-30b-5p/hsa-miR-374b-5p/hsa-miR-99b-5p |
| **Osteoclast differentiation** | 3.12E-04 | hsa-let-7c-5p/hsa-miR-100-5p/hsa-miR-126-5p/hsa-miR-141-3p/hsa-miR-182-5p/hsa-miR-192-5p/hsa-miR-194-5p/hsa-miR-199a-5p/hsa-miR-200a-3p/hsa-miR-200b-3p/hsa-miR-200c-3p/hsa-miR-204-5p/hsa-miR-223-3p/hsa-miR-28-5p/hsa-miR-30b-5p/hsa-miR-374b-5p/hsa-miR-532-5p |
| **Regulation of actin cytoskeleton** | 3.12E-04 | hsa-let-7c-5p/hsa-miR-100-5p/hsa-miR-126-5p/hsa-miR-141-3p/hsa-miR-182-5p/hsa-miR-183-5p/hsa-miR-192-5p/hsa-miR-194-5p/hsa-miR-199a-5p/hsa-miR-200a-3p/hsa-miR-200b-3p/hsa-miR-200c-3p/hsa-miR-204-5p/hsa-miR-223-3p/hsa-miR-27a-5p/hsa-miR-28-5p/hsa-miR-30b-5p |
| **Toxoplasmosis** | 3.12E-04 | hsa-let-7c-5p/hsa-miR-100-5p/hsa-miR-141-3p/hsa-miR-182-5p/hsa-miR-183-5p/hsa-miR-192-5p/hsa-miR-194-5p/hsa-miR-199a-5p/hsa-miR-200a-3p/hsa-miR-200b-3p/hsa-miR-200c-3p/hsa-miR-204-5p/hsa-miR-215-5p/hsa-miR-223-3p/hsa-miR-28-5p/hsa-miR-30b-5p/hsa-miR-374b-5p |
| **Gastric cancer** | 3.17E-04 | hsa-let-7c-5p/hsa-miR-100-5p/hsa-miR-126-5p/hsa-miR-141-3p/hsa-miR-182-5p/hsa-miR-183-5p/hsa-miR-192-5p/hsa-miR-194-5p/hsa-miR-199a-5p/hsa-miR-200a-3p/hsa-miR-200b-3p/hsa-miR-200c-3p/hsa-miR-204-5p/hsa-miR-215-5p/hsa-miR-223-3p/hsa-miR-27a-5p/hsa-miR-28-5p/hsa-miR-30b-5p/hsa-miR-374b-5p/hsa-miR-532-5p/hsa-miR-99b-5p |
| **Platelet activation** | 3.17E-04 | hsa-let-7c-5p/hsa-miR-100-5p/hsa-miR-141-3p/hsa-miR-182-5p/hsa-miR-183-5p/hsa-miR-192-5p/hsa-miR-194-5p/hsa-miR-199a-5p/hsa-miR-200a-3p/hsa-miR-200b-3p/hsa-miR-200c-3p/hsa-miR-204-5p/hsa-miR-28-5p/hsa-miR-374b-5p/hsa-miR-532-5p |
| **Pancreatic cancer** | 3.17E-04 | hsa-let-7c-5p/hsa-miR-100-5p/hsa-miR-126-5p/hsa-miR-141-3p/hsa-miR-182-5p/hsa-miR-183-5p/hsa-miR-192-5p/hsa-miR-194-5p/hsa-miR-199a-5p/hsa-miR-200a-3p/hsa-miR-200b-3p/hsa-miR-200c-3p/hsa-miR-204-5p/hsa-miR-215-5p/hsa-miR-223-3p/hsa-miR-27a-5p/hsa-miR-28-5p/hsa-miR-30b-5p/hsa-miR-374b-5p/hsa-miR-99b-5p |
| **Longevity regulating pathway - multiple species** | 3.47E-04 | hsa-let-7c-5p/hsa-miR-100-5p/hsa-miR-141-3p/hsa-miR-182-5p/hsa-miR-183-5p/hsa-miR-192-5p/hsa-miR-194-5p/hsa-miR-199a-5p/hsa-miR-200a-3p/hsa-miR-200c-3p/hsa-miR-204-5p/hsa-miR-223-3p/hsa-miR-28-5p/hsa-miR-30b-5p/hsa-miR-374b-5p/hsa-miR-99b-5p |
| **Fluid shear stress and atherosclerosis** | 3.47E-04 | hsa-let-7c-5p/hsa-miR-100-5p/hsa-miR-126-5p/hsa-miR-141-3p/hsa-miR-182-5p/hsa-miR-192-5p/hsa-miR-194-5p/hsa-miR-199a-5p/hsa-miR-200a-3p/hsa-miR-200b-3p/hsa-miR-200c-3p/hsa-miR-204-5p/hsa-miR-215-5p/hsa-miR-223-3p/hsa-miR-27a-5p/hsa-miR-28-5p/hsa-miR-30b-5p/hsa-miR-374b-5p |
| **Hepatocellular carcinoma** | 3.47E-04 | hsa-let-7c-5p/hsa-miR-100-5p/hsa-miR-126-5p/hsa-miR-141-3p/hsa-miR-182-5p/hsa-miR-183-5p/hsa-miR-192-5p/hsa-miR-194-5p/hsa-miR-199a-5p/hsa-miR-200a-3p/hsa-miR-200b-3p/hsa-miR-200c-3p/hsa-miR-204-5p/hsa-miR-215-5p/hsa-miR-223-3p/hsa-miR-27a-5p/hsa-miR-28-5p/hsa-miR-30b-5p/hsa-miR-374b-5p/hsa-miR-532-5p/hsa-miR-99b-5p |
| **Ubiquitin mediated proteolysis** | 3.47E-04 | hsa-miR-141-3p/hsa-miR-182-5p/hsa-miR-183-5p/hsa-miR-192-5p/hsa-miR-194-5p/hsa-miR-199a-5p/hsa-miR-200a-3p/hsa-miR-200b-3p/hsa-miR-200c-3p/hsa-miR-204-5p/hsa-miR-215-5p/hsa-miR-223-3p/hsa-miR-30b-5p |
| **ErbB signaling pathway** | 3.64E-04 | hsa-let-7c-5p/hsa-miR-100-5p/hsa-miR-126-5p/hsa-miR-141-3p/hsa-miR-182-5p/hsa-miR-183-5p/hsa-miR-192-5p/hsa-miR-194-5p/hsa-miR-199a-5p/hsa-miR-200a-3p/hsa-miR-200b-3p/hsa-miR-200c-3p/hsa-miR-204-5p/hsa-miR-223-3p/hsa-miR-27a-5p/hsa-miR-28-5p/hsa-miR-374b-5p/hsa-miR-99b-5p |
| **Phospholipase D signaling pathway** | 3.64E-04 | hsa-let-7c-5p/hsa-miR-100-5p/hsa-miR-141-3p/hsa-miR-182-5p/hsa-miR-192-5p/hsa-miR-199a-5p/hsa-miR-200a-3p/hsa-miR-200b-3p/hsa-miR-200c-3p/hsa-miR-204-5p/hsa-miR-27a-5p/hsa-miR-28-5p/hsa-miR-30b-5p/hsa-miR-374b-5p/hsa-miR-532-5p/hsa-miR-99b-5p |
