# Supplementary material for: Postprandial transfer of colostral extracellular vesicles and their protein and miRNA cargo in neonatal calves
Source: PLoS One. 2020 Feb 28;15(2):e0229606. doi: 10.1371/journal.pone.0229606 (PMC7048281; doi:10.1371/journal.pone.0229606)
Supplement: Attachment — Submitted filename: Response to Reviewers.docx [file pone.0229606.s008.docx]

Response to Reviewers

PONE-D-19-23560
Postprandial transfer of colostral extracellular vesicles and their protein and miRNA cargo in neonatal calves
PLOS ONE

Dear Editors, Dear Reviewers,

We would like to thank you for the helpful comments and extensive review! Please find our replies to all concerns raised below in red directly in the text.

Sincerely Benedikt Kirchner

Journal Requirements:

When submitting your revision, we need you to address these additional requirements.

1. Please ensure that your manuscript meets PLOS ONE's style requirements, including those for file naming. The PLOS ONE style templates can be found at <http://www.journals.plos.org/plosone/s/file?id=wjVg/PLOSOne_formatting_sample_main_body.pdf> and <http://www.journals.plos.org/plosone/s/file?id=ba62/PLOSOne_formatting_sample_title_authors_affiliations.pdf>

Headings, legends and file names have been modified to follow PLOS ONE naming conventions.

2. In your Methods section, please include a comment about the state of the animals following this research. Were they euthanized or housed for use in further research? If any animals were sacrificed by the authors, please include the method of euthanasia and describe any efforts that were undertaken to reduce animal suffering.

We included the mentioned comment in the Methods section on line 143.

3. We note that you are reporting an analysis of a microarray, next-generation sequencing, or deep sequencing data set. PLOS requires that authors comply with field-specific standards for preparation, recording, and deposition of data in repositories appropriate to their field. Please upload these data to a stable, public repository (such as ArrayExpress, Gene Expression Omnibus (GEO), DNA Data Bank of Japan (DDBJ), NCBI GenBank, NCBI Sequence Read Archive, or EMBL Nucleotide Sequence Database (ENA)). In your revised cover letter, please provide the relevant accession numbers that may be used to access these data. For a full list of recommended repositories, see <http://journals.plos.org/plosone/s/data-availability#loc-omics> or <http://journals.plos.org/plosone/s/data-availability#loc-sequencing>.

We changed the availability of our data deposited on ENA to public prior to publication to allow for access of all sequencing raw data including the newly added data set. All files can be found under the original accession number PRJEB28002 as mentioned in line 205.

4. We note that you have included the phrase “data not shown” in your manuscript. Unfortunately, this does not meet our data sharing requirements. PLOS does not permit references to inaccessible data. We require that authors provide all relevant data within the paper, Supporting Information files, or in an acceptable, public repository. Please add a citation to support this phrase or upload the data that corresponds with these findings to a stable repository (such as Figshare or Dryad) and provide and URLs, DOIs, or accession numbers that may be used to access these data. Or, if the data are not a core part of the research being presented in your study, we ask that you remove the phrase that refers to these data.

Since our reasoning to pool fractions from sucrose density gradient can also be understood from data already included in the manuscript, we removed our mentioning of preliminary results and instead linked them to supplemental table 1. The corresponding sentence from line 160 now reads:

“Sucrose gradient fractions of 40 % and 50 %, corresponding to a density of 1.1764 g/ml and 1.2296 g/ml, respectively, were pooled to increase yield, as results showed these to be most enriched in colostrum EVs (S1 Table).”

5.  PLOS ONE now requires that authors provide the original uncropped and unadjusted images underlying all blot or gel results reported in a submission’s figures or Supporting Information files. This policy and the journal’s other requirements for blot/gel reporting and figure preparation are described in detail at <https://journals.plos.org/plosone/s/figures#loc-blot-and-gel-reporting-requirements> and <https://journals.plos.org/plosone/s/figures#loc-preparing-figures-from-image-files>. When you submit your revised manuscript, please ensure that your figures adhere fully to these guidelines and provide the original underlying images for all blot or gel data reported in your submission. See the following link for instructions on providing the original image data: <https://journals.plos.org/plosone/s/figures#loc-original-images-for-blots-and-gels>.

In your cover letter, please note whether your blot/gel image data are in Supporting Information or posted at a public data repository, provide the repository URL if relevant, and provide specific details as to which raw blot/gel images, if any, are not available. Email us at [plosone@plos.org](mailto:plosone@plos.org) if you have any questions.

We added raw images of all blots used for Fig 4 including the newly added HSP70 in the supporting information as stated by the PLOS ONE guidelines (S1_raw_images.pdf).

Additional Editor Comments (if provided):

Please, add page and line numbers

Page and line numbers were added.

As emphasized by the referee, this study is susceptible to have a strong impact in a highly controversial field. Therefore, it is necessary to point some limitations of the experimental approach, for example the fact that colostral exosomes differ substantiality from mature milk exosomes or the fact that we have almost no data on the kinetics of colostral exosome uptake.

We expanded our introduction and discussion to cover all mentioned limitations (see line 107 and 702).

As suggested by the referee, it would be good to precise in the Materials and Methods section that the upper fat layer of the colostrum is rich in microRNAs. This fraction is not taken in account in Fig.7 although it may contribute to the profile of miRNAs in calf plasma and even calf plasma EVs.

We emphasized the removal of the upper fat layer in the Material and Methods section on lines 148 and 152. Furthermore, we extended our discussion about the limitations of this study to include the role of fat associated miRNAs (line 608).

The referee also rightly suggests providing in as much as possible absolute copy numbers of microRNAs instead of relative abundance.

A table of raw copy numbers for all significantly regulated miRNA was added to the supporting information (S4 Table).

The biochemical characterization of the exosomes is based on the detection of CD63 and the absence of calnexine. As proposed by the refere, it would be nice do I have additional markers like CD9 and CD81.

We added HSP70 as a cytosolic marker for exosomes as suggested by the MISEV guidelines in 2018. Unfortunately, we have no established antibody assay for CD9 or CD81 in bovines yet.

The legend of figure 5 is rather obscure. If I am not mistaken, there is no colostrum sample in this figure although the legend mentions “colostrum or plasma” (line 6).

That is absolutely right. The misleading mention of colostrum was removed.

One minor point. Page 5, line 15 : I wonder whether one should read “solely for 40-50% SDG” instead of “solely from…”.

We agree that “solely for” makes more sense and changed the sentence accordingly.


Reviewers' comments:

Reviewer's Responses to Questions

**Comments to the Author**

1. Is the manuscript technically sound, and do the data support the conclusions?

The manuscript must describe a technically sound piece of scientific research with data that supports the conclusions. Experiments must have been conducted rigorously, with appropriate controls, replication, and sample sizes. The conclusions must be drawn appropriately based on the data presented.

Reviewer #1: No

2. Has the statistical analysis been performed appropriately and rigorously?

Reviewer #1: No

3. Have the authors made all data underlying the findings in their manuscript fully available?

The [PLOS Data policy](http://www.plosone.org/static/policies.action#sharing) requires authors to make all data underlying the findings described in their manuscript fully available without restriction, with rare exception (please refer to the Data Availability Statement in the manuscript PDF file). The data should be provided as part of the manuscript or its supporting information, or deposited to a public repository. For example, in addition to summary statistics, the data points behind means, medians and variance measures should be available. If there are restrictions on publicly sharing data—e.g. participant privacy or use of data from a third party—those must be specified.

Reviewer #1: No

4. Is the manuscript presented in an intelligible fashion and written in standard English?

PLOS ONE does not copyedit accepted manuscripts, so the language in submitted articles must be clear, correct, and unambiguous. Any typographical or grammatical errors should be corrected at revision, so please note any specific errors here.

Reviewer #1: Yes

5. Review Comments to the Author

Please use the space provided to explain your answers to the questions above. You may also include additional comments for the author, including concerns about dual publication, research ethics, or publication ethics. (Please upload your review as an attachment if it exceeds 20,000 characters)

Reviewer #1: Review manuscript PONE-D-19-23560


Postprandial transfer of colostral extracellular vesicles and their protein and miRNA cargo in neonatal calves
Benedikt Kirchner, Dominik Buschman, Vijay Paul, Michael W. Pfaffl

General comment
The uptake of bovine milk exosomes and their miR cargo into the systemic circulation of human milk consumers is still a matter of debate in dairy research and human medicine.
With the intention to bring more light into milk-mediated systemic miR communication, the authors used bovine colostrum as a potential model for maternal-neonatal miR transfer.
To elucidate the role of bovine colostrum extracellular vesicles (EVs) and exosomes and their RNA cargo, the investigators assessed the potential postprandial transfer of colostral EVs to the circulation of newborn calves by analysing colostrum-specific proteins and miRNAs, including specific isoforms (isomiRs) in cells, EV isolations and unfractionated samples from blood and colostrum. Postprandial blood samples of calves show a time-dependent
increase in EVs that share morphological and protein characteristics of colostral EVs. Surprisingly, analysis of miRNA expression profiles by Next-Generation Sequencing
gave a different picture. Although significant postprandial expression changes
could only be detected for calf EV samples, expression profiles show very limited
overlap with highly expressed miRNAs in colostral EVs or colostrum in general. The authors concluded that from their experimental data that the uptake of membrane-associated protein cargo but not luminal miRNAs from colostral EVs into the circulation of neonatal calves may be selective.

The reviewer is not convinced that the presented colostrum-EV-based conclusions apply to transfer situation of exosomes and their miR content of mature bovine milk in humans and non-ruminant animal models. It should be emphasized that colostrum is a very unique mammary gland-derived secretory product.
Milk EVs derive from two independent secretory pathways: (1) the Golgi-endoplasmic reticulum-derived pathway common to milk fat globule secretion and (2) the endosomal multivesicular body pathway of exosome secretion (Reinhardt TA, Lippolis JD, Nonnecke BJ, Sacco RE. Bovine milk exosome proteome. J Proteomics. 2012;75(5):1486-92; Benmoussa A, Gotti C, Bourassa S, Gilbert C, Provost P. Identification of protein markers for extracellular vesicle (EV) subsets in cow's milk. J Proteomics. 2019;192:78-88).

The distinction between origins of different milk EV populations is a valuable addition to our discussion. We therefore expanded and corrected our discussion to include this fact and to give another possible conclusion for the disparate uptake of miRNA and proteins (line 688).

The mean diameter of EV particles in the range of 130-160 nm clearly indicates that the investigators primarily followed the take up of non-exosome EVs, because purified bovine and equine milk exosomes have reported diameters of 50-100 nm and 30-100 nm (Reinhardt TA, Lippolis JD, Nonnecke BJ, Sacco RE. Bovine milk exosome proteome. J Proteomics. 2012;75(5):1486-92; Sedykh SE, Purvinish LV, Monogarov AS, Burkova EE, Grigor'eva AE, Bulgakov DV, Dmitrenok PS, Vlassov VV, Ryabchikova EI, Nevinsky GA. Purified horse milk exosomes contain an unpredictable small number of major proteins. Biochim Open. 2017;4:61-72).

Differences in size measured by TEM (as in the mentionend papers by Reinhardt et al and Sedykh et al) and NTA have been observed in a number of studies and are caused mainly by two factors: 1. The detection limit of small particles in TEM is significantly lower as for NTA (~50 - 70nm). This leads to a shift towards bigger mean sizes in NTA that are slightly overestimated. A better measuring variable to compare sizes between TEM and NTA would therefore be the modal diameter (we changed line 542 accordingly to avoid misinterpretation), which represents the predominant vesicle size and is much closer to sizes described by Reinhardt et al and Sedykh et al (98.3 - 112.4nm). 2. TEM sample preparation may lead to shrinkage of vesicles due to chemical fixation and/or embedding and contrasting with methyl cellulose and uranyl acetate. Estimates for size reduction in TEM range between 12 - 21 %.

van der Pol E, Coumans FAW, Grootemaat AE, Gardiner C, Sargent IL, Harrison P, et al. Particle size distribution of exosomes and microvesicles determined by transmission electron microscopy, flow cytometry, nanoparticle tracking analysis, and resistive pulse sensing. J Thromb Haemost. 2014; 12: 1182–1192. doi: 10.1111/jth.12602.

Jensen OA, Prause JU, Laursen H. Shrinkage in preparatory steps for SEM. A study on rabbit corneal endothelium. Albrecht Von Graefes Arch Klin Exp Ophthalmol. 1981; 215: 233–242. doi: 10.1007/bf00407662.

While we agree, that our EV preparations most likely contain a large number of non-exosomal EVs, we do not think that the size distributions measured in this study, are a valid indicator that we primarily followed the take up of non-exosomes EVs. Taken into account the limitations of both TEM and NTA, we believe that our EV preparations are well within the size range of “classical” exosomes.

Moreover, the postprandial increase of BTN1A1 (butyrophilin) and lactadherin (MFGE8), predominant proteins of the MFG membrane (Ogg SL, Weldon AK, Dobbie L, Smith AJ, Mather IH. Expression of butyrophilin (Btn1a1) in lactating mammary gland is essential for the regulated secretion of milk-lipid droplets. Proc Natl Acad Sci U S A. 2004;101(27):10084-9. Robenek H, Hofnagel O, Buers I, Lorkowski S, Schnoor M, Robenek MJ, Heid H, Troyer D, Severs NJ. Butyrophilin controls milk fat globule secretion. Proc Natl Acad Sci U S A. 2006;103(27):10385-10390; Reinhardt TA, Lippolis JD, Nonnecke BJ, Sacco RE. Bovine milk exosome proteome. J Proteomics. 2012;75(5):1486-92), underline the preferred uptake of MFG-related non-exosomal EVs. Compared with the MFG membrane proteome, a 15–30-fold reduction in the abundance of xanthine oxidase, butyrophilin, lactadherin/MGFE8 and adipophilin/perilipin-2 has been observed in milk exosome membranes. (Reinhardt TA, Lippolis JD, Nonnecke BJ, Sacco RE. Bovine milk exosome proteome. J Proteomics. 2012;75(5):1486-92).

The referee rightly pointed out that BTN1A1 and MFGE8 are enriched in the MFG membrane. We concur that the postprandial increase of these proteins is underlining a preferred uptake of non-exosomal EVs. However, since colostrum EV preparations display high expression of CD63 and HSP70 as well, two proteins absent from MFGM proteomes and the fact that postprandial expression patterns for CD63 and HSP70 are showing increasing levels similar to BTN1A1 and MFGE8, we cannot rule out transfer of exosomal colostrum EVs. Our reasoning for choosing BTN1A1 and MFGE8 as indicators for a general transfer of colostral EVs, is their milk specific expression especially when compared to the milk exosomal proteome which is mostly enriched in proteins that can be found in almost all exosomal preparations no matter the tissue or biofluid of origin.

Reinhardt TA, Lippolis JD, Nonnecke BJ, Sacco RE. Bovine milk exosome proteome. J Proteomics. 2012; 75: 1486–1492. doi: 10.1016/j.jprot.2011.11.017.

Reinhardt TA, Lippolis JD. Bovine milk fat globule membrane proteome. J Dairy Res. 2006; 73: 406–416. doi: 10.1017/S0022029906001889.

The concomitant presence of the three tetraspanins CD9, CD63 and CD81 was suggested to characterize an EV subset as exosomes (Kowal J, Arras G, Colombo M, Jouve M, Morath JP, Primdal-Bengtson B, Dingli F, Loew D, Tkach M, Théry C. Proteomic comparison defines novel markers to characterize heterogeneous populations of extracellular vesicle subtypes. Proc Natl Acad Sci U S A. 2016;113(8):E968-77). Furthermore, it has been recommended to determine TSG101 as an exosomal marker protein of cow´s milk (Koh YQ, Peiris HN, Vaswani K, Meier S, Burke CR, Macdonald KA, Roche JR, Almughlliq F, Arachchige BJ, Reed S, Mitchell MD. Characterization of exosomes from body fluids of dairy cows. J Anim Sci. 2017;95(9):3893-3904; Benmoussa A, Gotti C, Bourassa S, Gilbert C, Provost P. Identification of protein markers for extracellular vesicle (EV) subsets in cow's milk. J Proteomics. 2019;192:78-88). A deficit of their study is the fact that the authors only used CD63 as a potential exosome marker.

We agree that our EV characterization would greatly benefit from a second EV marker. Unfortunately, we have no established antibodies for further tetraspanins or TSG101 in bovines. We therefore chose HSP70 as a positive, cytosolic marker for EVs as proposed by the MISEV guidelines to demonstrate purity of EV preparations and changed lines 293, 337, 348 and 578 accordingly.

Witwer KW, Soekmadji C, Hill AF, Wauben MH, Buzás EI, Di Vizio D, et al. Updating the MISEV minimal requirements for extracellular vesicle studies: building bridges to reproducibility. J Extracell Vesicles. 2017; 6: 1396823. doi: 10.1080/20013078.2017.1396823.

However, most critical is the collection of colostrum at the day of parturition. It has been demonstrated that there is a dramatic postnatal change of the colostrum exosome proteome compared to mature milk exosome proteome. It was observed that certain proteins that were highly abundant in 24 h colostrum exosomes, slowly diminished at 48 and 72 h and were at similar amounts to the mature milk exosome sample (Samuel M, Chisanga D, Liem M, Keerthikumar S, Anand S, Ang CS, Adda CG, Versteegen E, Jois M, Mathivanan S. Bovine milk-derived exosomes from colostrum are enriched with proteins implicated in immune response and growth. Sci Rep. 2017;7(1):5933). Early colostral exosomes are enriched with proteins implicated in immune response and intestinal cell proliferation and may not be comparable to mature milk exosomes in terms of composition, RNA content and physiological functions. Immediate postnatal exosomes are thus unique and not a representative model for mature human milk exosomes and their potential systemic beneficial and adverse effects in humans (Zempleni J, Aguilar-Lozano A, Sadri M, Sukreet S, Manca S, Wu D, Zhou F, Mutai E. Biological Activities of Extracellular Vesicles and Their Cargos from Bovine and Human Milk in Humans and Implications for Infants. J Nutr. 2017;147(1):3-10; Melnik BC, Schmitz G. Exosomes of pasteurized milk: potential pathogens of Western diseases. J Transl Med. 2019;17(1):3). Thus, the presented model using early colostrum exosomes is not transferable to the in vivo situation of exosome biology and exosome trafficking of exosomes of midlactation dairy cows. These limitations have to be outlined in the Section Limitations of this study. They are of crucial importance because the unexperienced reader may draw misleading conclusions for the in vivo exosome traffic of pasteurized commercial milk to the human milk consumer.

The reviewer raises a valid concern, in pointing out the differences between colostrum and milk derived EVs and their potential roles in physiology. By choosing newborn calves as an animal model, we thought to utilize its unique advantages (no prior diet whatsoever, well-known uptake of other colostrum components,…) to gain more insight in EV transfer and stayed intentionally away from midlactation milk and adult humans. We agree that this might be misleading an unexperienced reader to reach the wrong conclusions and will gladly expand our introduction section to emphasize this (line 107)

Another concern regarding the study design is the fact that the authors provided no kinetic studies of exosome or miR uptake. Baier et al. (see ref. 30) demonstrated that the maximum of miR-29b uptake in human volunteers 6 hours after oral intake of commercial milk. The calf is a ruminant and the reviewer is not aware of available data on colostrum and milk exosome kinetics in this species. It is not known which part of the complex bovine intestine is the major area for exosome uptake. Thus, the maximum of colostral exosome uptake and miR appearance in the calf blood plasma is an uncertainty that should be emphasized.

We emphasized the uncertainty of colostral EV uptake in kinetics in the discussion (line 702)


Specific comments

Introduction
3rd paragraph
The data of Howard et al. demonstrate that the majority of milk miR-29b (nearly two thirds) survive pasteurization, homogenization and cooled storage (see ref. 22). This is in agreement with the data reported for pasteurized cow´s milk by Golan-Gerstl et al. (Golan-Gerstl R, Elbaum Shiff Y, Moshayoff V, Schecter D, Leshkowitz D, Reif S. Characterization and biological function of milk-derived miRNAs. Mol Nutr Food Res. 2017;61(10):1700009).

While we consider a reduction by over one third to be significant, we changed line 74 to better reflect that the majority of miRNA survive processing and storage. We also included Golan-Gerstl et al as an additional reference.

The study of Baier et al. (see ref. 30) is convincing for the reviewer and other researchers. The increase in miR-29b in blood monocytes 6 h after milk consumption can be plausibly explained by exosomal miR uptake. The Auerbach et al. study (see ref. 31) had a severe technical problem as exosome samples were not continuously cooled. The authors reported that the sample arrived with largely sublimated dry ice. Thus, exosome or exosome protein degradation could not be excluded.

We added a mention of this technical problem to line 84 to allow the reader to put the study of Auerbach et al in perspective.

The cited study of Title et al (see. ref. 32) was not a well-designed study and has been criticized by various independent researchers (Melnik BC, Kakulas F, Geddes DT, Hartmann PE, John SM, Carrera-Bastos P, Cordain L, Schmitz G. Milk miRNAs: simple nutrients or systemic functional regulators? Nutr Metab (Lond). 2016;13:42).

The arguments presented by Melnik et al are not convincing to us in discrediting the study of Title et al. While it has been shown that miRNAs can have effects in very low concentrations of femto- to picomolar range, Title et al reported an estimated yield of less than 3 copies/cell for possibly transferred miRNAs and therefore well below that range. Melnik et al furthermore claim that the absence of an essential sorting motif in mir-375 ({(A/U)(C2-4)(A/U)}, citing an abstract presented by Shurtleff et al in 2015 on a conference in San Diego), should prevent packaging of mir-375 into exosomes. While the same motif is also missing from well known milk-EV associated mature miRNAs like mir-148a and even mir-200a/b/c, Shurtleff et al themselves report in their paper on the same topic in 2016, that they could not identify any significant primary sequence motifs for miRNAs. Lastly, Melnik et al argue that the knock-out of mir-375 or mir-200c, leads to a severe dysregulation of endocytosis and thus preventing uptake of orally administered milk-EV associated miRNAs. While there is evidence for mir-375 to affect endocytosis in insulin-secreting and cancer cells, we see no proof that this would lead to a complete shutdown of EV miRNA cargo uptake. Similar to this mir-200c down-regulates KRas, which is a positive regulator in endo- and exocytosis.

Shurtleff MJ, Temoche-Diaz MM, Karfilis KV, Ri S, Schekman R. Y-box protein 1 is required to sort microRNAs into exosomes in cells and in a cell-free reaction. Elife. 2016; 5. doi: 10.7554/eLife.19276.

The authors should note that bovine milk exosomes reached distant organs in independent studies in mice (Arntz OJ, Pieters BC, Oliveira MC, Broeren MG, Bennink MB, de Vries M, van Lent PL, Koenders MI, van den Berg WB, van der Kraan PM, van de Loo FA. Oral administration of bovine milk derived extracellular vesicles attenuates arthritis in two mouse models. Mol Nutr Food Res. 2015;59(9):1701-12; Manca S, Upadhyaya B, Mutai E, Desaulniers AT, Cederberg RA, White BR, Zempleni J. Milk exosomes are bioavailable and distinct microRNA cargos have unique tissue distribution patterns. Sci Rep. 2018;8(1):11321)

In contrast to the presented skepticism concerning systemic milk exosome traffic, pharmacologists regard milk exosomes as ideal carriers for systemic drug transfer because milk exosomes exhibit cross-species tolerance with no adverse immune and inflammatory response and reach distant target tissue (Munagala R, Aqil F, Jeyabalan J, Gupta RC. Bovine milk-derived exosomes for drug delivery. Cancer Lett. 2016;371(1):48-61; Betker JL, Angle BM, Graner MW, Anchordoquy TJ. The Potential of Exosomes From Cow Milk for Oral Delivery. J Pharm Sci. 2019;108(4):1496-1505). Notably, orally delivered paclitaxel (PAC)-loaded exosomes (ExoPAC) showed significant tumor growth inhibition against human lung tumor xenografts in nude mice, demonstrating a systemic effect of milk exosomes (Agrawal AK, Aqil F, Jeyabalan J, Spencer WA, Beck J, Gachuki BW, Alhakeem SS, Oben K, Munagala R, Bondada S, Gupta RC. Milk-derived exosomes for oral delivery of paclitaxel. Nanomedicine. 2017;13(5):1627-1636). Pharmaceutical industry such as Roche would not invest $ 1 Billion for milk exosome preparations for systemic drug delivery (<https://www.genengnews.com/topics/omics/got-milk-roche-to-apply-puretech-exosomes-platform-in-1b-collaboration/>). Thus, overwhelming evidence supports the systemic traffic and long-distance effects of bovine exosomes derived from commercial milk in humans and non-ruminant animal models.

Therefore, the reviewer disagrees with the authors´ statement that “uptake of milk-derived EVs is more likely to be observed between mother and direct offspring as opposed to a cross-species transfer to adult individuals”.

We agree that the original statement might be misleading in that it is making the transfer of orally administered milk EVs in adults sounding less plausible. That was not our intention. We changed line 100 accordingly.

In contrast to Witwer et al. (see ref. 36), the majority of independent international milk exosome researchers (USA, Canada, Australia, India and Germany) accept the bioavailability and systemic effects of bovine milk exosomes (Manca S, Upadhyaya B, Mutai E, Desaulniers AT, Cederberg RA, White BR, Zempleni J. Milk exosomes are bioavailable and distinct microRNA cargos have unique tissue distribution patterns. Sci Rep. 2018;8(1):11321; Wang L, Sadri M, Giraud D, Zempleni J. RNase H2-Dependent Polymerase Chain Reaction and Elimination of Confounders in Sample Collection, Storage, and Analysis Strengthen Evidence That microRNAs in Bovine Milk Are Bioavailable in Humans. J Nutr. 2018;148(1):153-159. Zempleni J, Sukreet S, Zhou F, Wu D, Mutai E. Milk-derived exosomes and metabolic regulation. Annu Rev Anim Biosci. 2019; 7:245-62; Benmoussa A, Provost P. Milk microRNAs in health and disease. Compr Rev Food Sci Food Safety. 2019; doi: 10.1111/1541-4337.12424; Melnik BC, Kakulas F, Geddes DT, Hartmann PE, John SM, Carrera-Bastos P, Cordain L, Schmitz G. Milk miRNAs: simple nutrients or systemic functional regulators? Nutr Metab (Lond). 2016;13:42; Agrawal AK, Aqil F, Jeyabalan J, Spencer WA, Beck J, Gachuki BW, Alhakeem SS, Oben K, Munagala R, Bondada S, Gupta RC. Milk-derived exosomes for oral delivery of paclitaxel. Nanomedicine. 2017;13(5):1627-1636).

We agree that there are multiple valid studies showing the systemic effect of orally delivered EVs. We originally focused our introduction on studies that highlighted transfer of EV associated miRNAs, but we can see that this maybe painted a rather too negative picture of dietary EV uptake in general. We added to the third paragraph of the introduction to correct that (line 89).

Material and Methods

Sample collection
As already outlined under general comments, early colostrum EVs and exosomes appear to represent a very unique composition to manage early stages of postnatal maturation and development of the newborn mammal. Early colostral exosomes differ substantially from mature milk exosomes which are the components of concern for human health (Melnik BC, Schmitz G. Exosomes of pasteurized milk: potential pathogens of Western diseases. J Transl Med. 2019;17(1):3).

The time points for calf plasma sampling are not based on available kinetic data of colostrum exosome uptake. It might be possible that the peak of maximal uptake lays in the interval between 6 h and 9-12 h, and may be missed in this study. The ruminant situation may change exosome uptake characteristics.

The investigators removed the fat layer, which is a major source of miRs, which thus escaped detection and follow up in this study.

We expanded our discussion to include the potential effect of fat-associated miRNAs and emphasized the removal of the fat layer in the Material and Methods section (line 148, 152 and 609).

The authors use the term “skim milk” throughout their manuscript, which is not correct: they analyzed “skimmed colostrum”, which should be used.

We changed all occurrences of skim milk to skimmed colostrum.

The authors state that sucrose gradient fractions of 40 % and 50 %, corresponding to a density of 1.1764 g/ml and 1.2296 g/ml, respectively, were pooled to increase yield, as preliminary results had shown these to be most enriched in colostrum EVs (data not shown).
These data are most important and should be provided.

Since our reasoning to pool fractions from sucrose density gradient can also be understood from data already included in the manuscript, we removed our mentioning of preliminary results and instead linked them to supplemental table 1. The corresponding sentence from line 160 now reads:

“Sucrose gradient fractions of 40 % and 50 %, corresponding to a density of 1.1764 g/ml and 1.2296 g/ml, respectively, were pooled to increase yield, as results showed these to be most enriched in colostrum EVs (S1 Table).”

The authors only rely on EV separation by density gradient centrifugation and ultracentrifugation. There is no control by other methods such as ExoQuick precipitation. Yamada et al. reported that the highest yield of exosomes was achieved using ultracentrifugation with ExoQuick precipitation (Yamada T, Inoshima Y, Matsuda T, Ishiguro N. Comparison of methods for isolating exosomes from bovine milk. J Vet Med Sci. 2012;74(11):1523-5). Thus, the investigators may have lost exosomes in their preparations.

Results
RNA analysis and bioinformatics

The major concern of this study is the fact that the authors do not provide absolute concentrations of their miR determinations but instead provide relative changes (delta values). Furthermore, there are no data for sensitivity and confidence intervals. Genomic mapping would be helpful to interpret the origin of the detected RNA sequences. A critical look to the data shows that regulatory miRs have been detected in EVs including tRNA, short regulatory RNAs, long non-coding RNAs and precursor-sequences of miRs.
From the point of particle energetics, it is not conceivable that exosome proteins and their miR content are disintegrated and send to different metabolic of signaling routes.

Therefore, the revised version of this manuscript should provide absolute data of miRs including a revision of corresponding figures and tables.

A table of raw copy numbers for all significantly regulated miRNA was added to the supporting information (S4 Table). No fold changes were mentioned in any figures or tables of the main manuscript.

Discussion
The revised version of this paper should emphasize the outlined limitations of their study.

Taken together, in the present form, this paper does not allow an approximation of the proportions of colostrum EVs of exosomal versus non-exosomal origin. The miR-enriched density fraction is obviously a mixture of denser non-exosomal EVs and smaller CD63-positive colostrum exosomes. Unfortunately, the investigators used CD63 as the only exosome marker. The reviewer agrees to the conceptual idea of partly repackaging of non-exosomal EVs in intestinal mucosa cell, but not for exosomes, which are able to pass intestinal and endothelial cell boundaries.

6. PLOS authors have the option to publish the peer review history of their article ([what does this mean?](https://journals.plos.org/plosone/s/editorial-and-peer-review-process#loc-peer-review-history)). If published, this will include your full peer review and any attached files.


If you choose “no”, your identity will remain anonymous but your review may still be made public.


**Do you want your identity to be public for this peer review?** For information about this choice, including consent withdrawal, please see our [Privacy Policy](https://www.plos.org/privacy-policy).

Reviewer #1: No


[NOTE: If reviewer comments were submitted as an attachment file, they will be attached to this email and accessible via the submission site. Please log into your account, locate the manuscript record, and check for the action link "View Attachments". If this link does not appear, there are no attachment files to be viewed.]
